# A Text-Book of Materia Medica, Therapeutics and Pharmacology

**Published:** 1900-03

**Authors:** 


A TEXT-BOOK OF MATERIA MEDICA, THERAPEUTICS AND PHARMACOLOGY. By GEORGE FRANK BUTLER, Ph. G, M. D., Professor of Materia Medica and of Clinical Medicine in the College of Physicians and Surgeons, Medical Department, University of Illinois, Chicago; Professor of General Medicine and Diseases of the Digestive System, Chicago Clinical School; Attending Physician to Cook County Hospital, etc., etc Third edition, thoroughly revised. Handsome 8vo volume of 874 pages. Illustrated. Philadelphia: W. B. Saunders. 1899.

The appearance of several editions of Dr. Butler's book in rapid succession gives evidence of its practicability, and its value in this respect is of a kind that commands the appreciation not only of practitioners but of teachers and students. The author recognises the emphatic need of today in the line of his work—namely, a more thorough knowledge of drugs, their properties, preparation and powers.

These rudiments of pharmacology are systematically arranged for convenient study, separate sections being given to pharmaceutical preparations and processes of their manufacture and to prescription writing. Then in the text of the main part of the work incompatibles, synergists, doses, physiological action and poisonous effects are discussed in connection with the individual drugs and their uses.

We do not deplore the absence of the customary list of diseases and remedies applicable thereto, which forms so extensive a part of many books on therapeutics, but which is a veritable *pons asinorum* to superficial students and practitioners. Whatever value such a list would possess is compensated for in the book before us by a clinical index which directs to the application of any drug in the various diseases. We desire to commend the author's adherence to pharmacopeial titles and definitions throughout work and the constant recognition of the resources furnished, in the direction of medicinal combinations, by the U.S. Pharmacopeia and the National Formulary. We could wish, however, that he had gone a step further and in the expression of doses given the metric system first place, recognising the old system only parenthetically.

On the whole, the volume furnishes one of our most complete and practical text-books on materia medica. E. H. L.

